# RNA pre-amplification enables large-scale RT-qPCR gene-expression studies on limiting sample amounts

**DOI:** 10.1186/1756-0500-2-235

**Published:** 2009-11-25

**Authors:** Joëlle Vermeulen, Stefaan Derveaux, Steve Lefever, Els De Smet, Katleen De Preter, Nurten Yigit, Anne De Paepe, Filip Pattyn, Frank Speleman, Jo Vandesompele

**Affiliations:** 1 Center for Medical Genetics, Ghent University Hospital, Ghent, Belgium; 2 Biogazelle, Ghent, Belgium

## Abstract

**Background:**

The quantitative polymerase chain reaction (qPCR) is a widely utilized method for gene-expression analysis. However, insufficient material often compromises large-scale gene-expression studies. The aim of this study is to evaluate an RNA pre-amplification method to produce micrograms of cDNA as input for qPCR.

**Findings:**

The linear isothermal Ribo-SPIA pre-amplification method (WT-Ovation; NuGEN) was first evaluated by measuring the expression of 20 genes in RNA samples from six neuroblastoma cell lines and of 194 genes in two commercially available reference RNA samples before and after pre-amplification, and subsequently applied on a large panel of 738 RNA samples extracted from neuroblastoma tumours. All RNA samples were evaluated for RNA integrity and purity. Starting from 5 to 50 nanograms of total RNA the sample pre-amplification method was applied, generating approximately 5 microgams of cDNA, sufficient to measure more than 1000 target genes. The results obtained from this study show a constant yield of pre-amplified cDNA independent of the amount of input RNA; preservation of differential gene-expression after pre-amplification without introduction of substantial bias; no co-amplification of contaminating genomic DNA; no necessity to purify the pre-amplified material; and finally the importance of good RNA quality to enable pre-amplification.

**Conclusion:**

Application of this unbiased and easy to use sample pre-amplification technology offers great advantage to generate sufficient material for diagnostic and prognostic work-up and enables large-scale qPCR gene-expression studies using limited amounts of sample material.

## Introduction

Amongst the various methods available to measure gene-expression, the reverse transcription quantitative polymerase chain reaction (RT-qPCR) is the most rapid, sensitive, and reproducible method [1-5]. However, it often remains challenging to obtain from clinical samples the amounts of mRNA required to perform a gene-expression analysis, especially for large-scale studies.

Therefore, it seems that a method capable of pre-amplifying nanogram quantities of RNA is essential, to ensure that sufficient material is available for high-throughput gene-expression profiling. Various pre-amplification methods have been proposed including as well PCR-based [6,7] as linear isothermal [8-10] pre-amplification strategies. Each method has proven to be effective in generating microgram quantities of cDNA from minute amounts of input RNA. While various studies have evaluated these methods for microarray-based procedures [11-17], only limited information is available for qPCR applications.

This paper extensively evaluates the linear isothermal Ribo-SPIA pre-amplification method for qPCR [10,18]. The method was first evaluated in RNA samples from neuroblastoma cell lines and commercially available reference RNA, and subsequently applied on a large panel of RNA samples extracted from neuroblastoma tumours, to be used in a prognostic multigene-expression signature study [19].

## Materials and methods

### Sample preparation

Total RNA was extracted from 6 neuroblastoma cell lines and 738 fresh frozen neuroblastoma tumour biopsies according to three methods in collaborating laboratories. Two commercial RNA samples were mixed (Universal Human Reference RNA (UHRR) from Stratagene and Human Brain Reference RNA (HBRR) from Ambion) to generate the four MAQC (MicroArray Quality Control) reference samples [20].

In order to assess the RNA purity and integrity, we performed a SPUD assay for the detection of enzymatic inhibitors [21] and a capillary gel electrophoresis analysis (Experion; Bio-Rad) to establish an RNA quality index (RQI).

### RNA pre-amplification and cDNA synthesis

Starting from 5, 15, or 50 ng of total RNA, the WT-Ovation RNA Pre-amplification method (NuGEN) was used according to the manufacturer's instructions, generating approximately 5 μg of cDNA [10,18].

In parallel the same RNA extracted from the neuroblastoma cell lines and the MAQC samples were used for conventional cDNA synthesis using the iScript cDNA Synthesis Kit according to the manufacturer's instructions (Bio-Rad).

### High-throughput real-time quantitative PCR based gene-expression

A qPCR assay was designed for each gene [Additional files 1, 2] and validated through an extensive analysis pipeline [22]. Real-time qPCR was performed in a 384-well-plate instrument (LC480, Roche).

See [Additional file 3] for more details on this section.

## Results

### Pre-amplification yield as a function of RNA input

**Figure 1:**
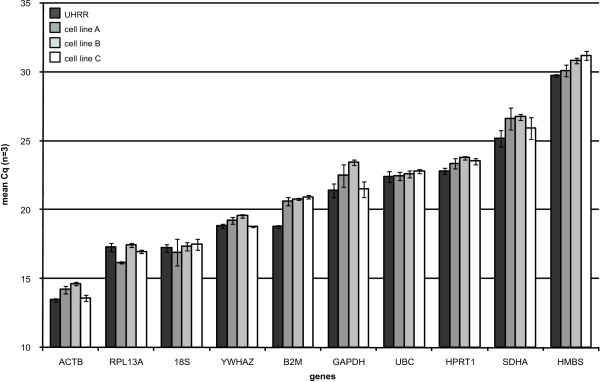
**Pre-amplification yield as a function of RNA input**. qPCR quantification (mean Cq of 3 pre-amplified samples starting from 5, 15 or 50 ng input RNA) of 10 reference genes in four pre-amplified samples (error bars denote standard deviation). The standard deviation on the mean Cq-value of the three pre-amplified products per sample is low indicating that the yield is not dependent on the amount of input RNA. UHRR: Universal Human Reference RNA.

In order to assess the influence of the amount of input RNA on the yield of pre-amplified cDNA we measured the expression of ten reference genes after pre-amplification starting from 5, 15 or 50 ng as input RNA from three cultured neuroblastoma cells and UHRR. Figure 1 shows that the method is highly reproducible and that the yield (as defined by the quantification cycle (Cq) value of commonly used reference genes) is not dependent on the amount of input RNA. For each gene (irrespective of the abundance level), the standard deviation on the mean Cq-value of the three pre-amplified products per sample is low (range 0.06 to 0.97, mean 0.30).

### Differential expression

**Figure 2:**
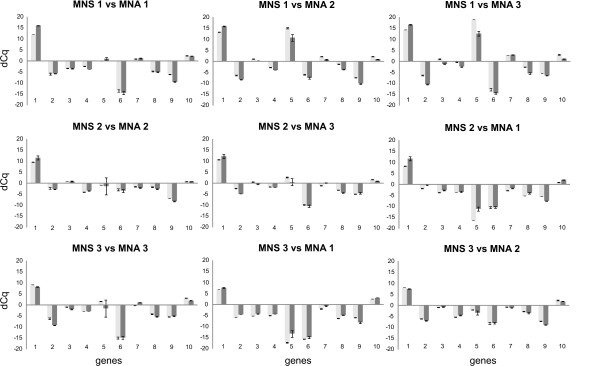
**Preservation of differential expression after pre-amplification**. Differential expression (expressed as difference in Cq or delta-Cq (dCq)) of 10 genes in three *MYCN *single copy (MNS) neuroblastoma cell lines (1: GI-ME-N; 2: SK-N-AS; 3: SK-N-SH) and three *MYCN *amplified (MNA) neuroblastoma cell lines (1: IMR-32; 2: N206; 3: NGP) before (light grey bars) and after sample pre-amplification (dark grey bars). The x-axis represents the differentially expressed genes. 1:*MYCN*; 2:*INHBA*; 3:*RGS4*; 4:*DKK3*; 5:*NTRK2*; 6:*TGFBI*; 7:*PMP22*; 8;*PLAT*; 9:*CMYC*; 10:*MTHFD2*. The dCq between MNS and MNA samples remains almost unchanged after pre-amplification indicating a preservation of differential expression.

**Figure 3:**
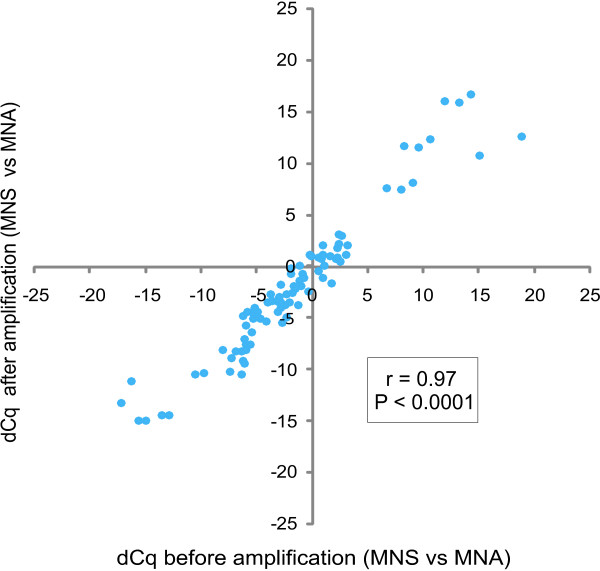
**Correlation of differential gene-expression before and after pre-amplification**. Correlation of differential gene-expression (expressed as difference in Cq or delta-Cq (dCq)) of ten genes in three *MYCN *single copy (MNS) and three *MYCN *amplified (MNA) neuroblastoma cell lines before (x-axis) and after pre-amplification (y-axis). Same data as in Figure 2. The observed correlation indicates preservation of differential expression after pre-amplification.

**Figure 4:**
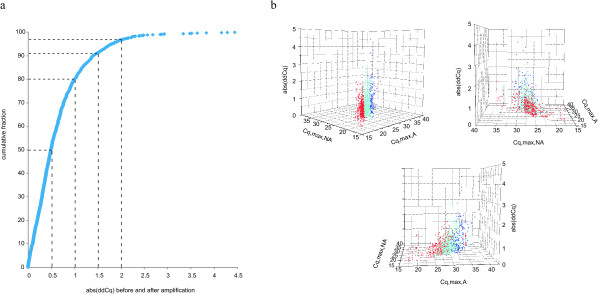
**Unbiased pre-amplification procedure**. a/Cumulative distribution plot of the absolute difference in delta-Cq-values (delta-delta-Cq or ddCq) before and after pre-amplification for 194 genes (12 reference genes and 182 targets of interest) in 100% Universal Human Reference RNA (UHRR) versus 100% Human Brain Reference RNA (HBRR) versus (25% UHRR + 75% HBRR) versus (75% UHRR + 25% HBRR). Each dot represents a ddCq-value between 2 samples before and after pre-amplification (in total 1164 data points). b/Three-dimensional representation of the ddCq (z-axis) versus the highest Cq-value amongst 4 values (2 replicates each of the 2 samples being compared before pre-amplification (Cq, max, NA: x-axis) and after pre-amplification (Cq, max, A: y-axis)). The lower the gene is expressed (high Cq-value), the higher the ddCq. Red dots: Cq, max, A - Cq, max, NA ≤ 2. Light blue dots: Cq, max, A - Cq, max, NA > 2; ≤ 5. Dark blue dots: Cq, max, A - Cq, max, NA > 5.

The pre-amplification method induces a recognized sequence-specific pre-amplification bias, meaning that some sequences or parts of transcripts pre-amplify better than others [Additional file 4, Figure S1]. However, most critically is the preservation of the differential expression levels between samples after pre-amplification. Using three *MYCN *single copy (MNS) and three *MYCN *amplified (MNA) neuroblastoma cell lines, we first measured the expression of 10 known differentially expressed genes (*MYCN *itself and nine known *MYCN *regulated genes [23]) before and after pre-amplification [Additional file 1]. The differential gene-expression (expressed as difference in Cq or delta-Cq (dCq) [Additional file 3 for an example]) between two samples remains equal after pre-amplification (Figure 2). We observed a high correlation between differential gene-expression of the ten genes between the MNS and MNA cell lines before and after pre-amplification (Spearman correlation coefficient: 96.7; P < 0.0001) (Figure 3). Next we measured the expression of a set of 194 genes (12 reference genes and 182 MAQC target genes) [20] before and after pre-amplification in the MAQC samples (replicates) [Additional file 2]. Quality control of the replicates showed that 83.3% of all replicates had a standard deviation <0.2; 96.0% <0.5; and 99.1% <1.0. Figure 4a shows that the difference in dCq (delta-delta-Cq or ddCq [Additional file 3 for an example]) before and after pre-amplification is less than 1 in 80.1%, less than 1.5 in 91.3% and less than 2 in 96.7% of the samples, indicating that the pre-amplification bias is acceptable. As shown in Figure 4b, the lower the gene is expressed (high Cq-value), the higher the ddCq, demonstrating that initial low expression and especially low expression after pre-amplification due to a lower pre-amplification efficiency for the region targeted with qPCR is associated with a higher bias.

### Pre-amplification method does not pre-amplify DNA

In order to determine if residual DNA in the RNA extract is co-amplified and consequently might confound the results, we pre-amplified pure human genomic DNA (HGDNA) and two RNA samples from neuroblastoma cell lines verified for absence of DNA and subsequently spiked with 1% and 10% HGDNA (2 ng DNA per 20 ng RNA input for pre-amplification) (Roche). We next performed qPCR with a DNA-specific primer pair (*NEUROD1*; RTPrimerDB ID 8113 [22]) and used HGDNA as positive control. No signal for *NEUROD1 *could be observed in the pre-amplified cell lines spiked with DNA as resulting DNA concentration after a 200× dilution of the pre-amplified product is lower than 0.5 pg/μl, which is below the detection level for qPCR. Moreover, the Cq-value of *NEUROD1 *was equal in the HGDNA that had undergone the above described pre-amplification procedure and in the HGDNA used as positive control. These results indicate that DNA is not co-amplified (data not shown).

### No need for purification of the pre-amplified products

To determine if purification of the pre-amplification product is required we performed a SPUD assay as described in [additional file 3] [21] and we compared the expression values of six reference genes using qPCR in 6 purified versus 6 non-purified pre-amplified neuroblastoma samples. The dCq between the negative control and both the purified and non-purified samples did not exceed 1, indicating absence of enzymatic inhibitors [Additional file 5, Figure S2]. PCR efficiencies were evaluated using two single curve efficiency algorithms (PCR Miner [24] and LinReg [25]) and were similar for both purified and non-purified samples, confirming the absence of enzymatic inhibitors in non-purified pre-amplified products [Additional file 6, Figure S3]. When using the same mass input amount of purified and non-purified products, we noticed on average 6.31 times (95% CI: 4.89 - 8.14) more amplifiable target in the purified products. This indicates that more than 80% of the non-purified mass consists of free dNTPs, primers and other molecules that are detected by spectrophotometric measurement and that there is a need for relatively more input for qPCR if non-purified pre-amplified material is used (Table 1).

**Table 1:** Expression values of 6 reference genes using qPCR in purified versus non-purified pre-amplified samples

		*UBC*	*GAPDH*	*RPL13A*	*YWHAZ*	*SDHA*	*HPRT1*	mean dCq	*2^dCq*
**sample 1**	**mean Cq NP1 (n = 2)**	**25.47**	**23.73**	**19.05**	**22.31**	**29.82**	**25.71**		
	**mean Cq P1 (n = 2)**	**21.54**	**22.19**	**15.87**	**18.29**	**25.94**	**22.03**		
	dCq	3.93	1.54	3.18	4.02	3.88	3.68	**3.37**	***10.34***

**sample 2**	**mean Cq NP2 (n = 2)**	**23.71**	**23.03**	**18.12**	**21.81**	**30.14**	**24.86**		
	**mean Cq P2 (n = 2)**	**20.95**	**21.54**	**15.63**	**18.53**	**28.14**	**21.89**		
	dCq	2.77	1.49	2.49	3.28	2.01	2.97	**2.5**	***5.65***

**sample 3**	**mean Cq NP3 (n = 2)**	**24.22**	**23.03**	**18.63**	**20.94**	**30.17**	**25.82**		
	**mean Cq P3 (n = 2)**	**21.1**	**21.75**	**16.04**	**17.66**	**28.67**	**22.72**		
	dCq	3.12	1.29	2.6	3.28	1.5	3.1	**2.48**	***5.57***

**sample 4**	**mean Cq NP4 (n = 2)**	**22.96**	**23.08**	**18.54**	**21.74**	**30.66**	**25.62**		
	**mean Cq P4 (n = 2)**	**19.69**	**21.89**	**16.07**	**18.63**	**28.83**	**22.48**		
	dCq	3.27	1.19	2.48	3.11	1.83	3.14	**2.5**	***5.66***

**sample 5**	**mean Cq NP5 (n = 2)**	**23.21**	**22.72**	**17.96**	**20.84**	**28.17**	**25.37**		
	**mean Cq P5 (n = 2)**	**20.64**	**21.22**	**15.6**	**17.83**	**25.73**	**22.11**		
	dCq	2.57	1.51	2.36	3.01	2.44	3.26	**2.52**	***5.74***

**sample 6**	**mean Cq NP6 (n = 2)**	**24.62**	**23.48**	**18.56**	**22.22**	**28.84**	**25.71**		
	**mean Cq P6 (n = 2)**	**21.52**	**21.87**	**15.91**	**19.03**	**26.97**	**22.67**		
	dCq	3.1	1.61	2.65	3.19	1.88	3.04	**2.58**	***5.96***

**average**									**6.31 (95% CI: 4.89 - 8.14)**

Cq: quantification cycle

dCq: difference in Cq or delta-Cq

NP: non-purified after pre-amplification

P: purified after pre-amplification

2^dCq designates the pre-amplifiable target ratio (purified/non-purified products)

In a last step of the evaluation of the necessity of pre-amplification clean-up, we measured the expression of ten reference genes in ten samples before and after pre-amplification. Comparison of the cumulative distribution plots of the ddCq-values obtained on purified and non-purified pre-amplified product showed that the plots almost completely overlap, providing further evidence that purification is not required [Additional file 7, Figure S4].

### Pre-amplification as a function of RNA quality

**Figure 5:**
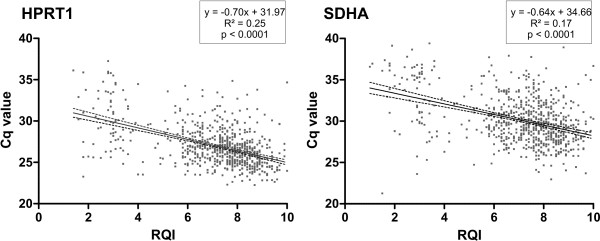
**Pre-amplification as a function of RNA quality**. Correlation between the detection levels of *HPRT1 *and *SDHA *and RNA Quality Index (RQI), in pre-amplified samples in which *HPRT1 *(n = 702) and *SDHA *(n = 699) were detectable (Cq < 40).

In order to assess the RNA quality of 738 neuroblastoma tumour samples, we performed a capillary gel electrophoresis analysis to establish an RQI. All samples were pre-amplified and qPCR was performed to measure the expression of two low abundant universally expressed reference genes (SDHA and HPRT1) [Additional file 8]. Both genes were undetectable in 22 (3.0%) samples, HPRT1 was undetectable in 14 (1.9%) additional samples, and SDHA in 17 (2.3%) additional samples. The average RQI was 2.7 (± 1.9 stdev) in the group of samples with missing value for at least one reference gene compared to 7.2 (± 1.7 stdev) in the group of samples where both reference genes were expressed (p < 0.0001). We found a negative correlation between the Cq-values of both reference genes and RQI (Figure 5).

## Discussion

An import limitation of gene-expression analysis in the current diagnostic workflow is the fact that often minimal amounts of biomaterial are procured. As such, in many cases only a few nanograms of total RNA are available. In order to measure a large number of genes on this limited material and to maximize the number of samples through collaborative studies, a robust sample pre-amplification method is required. In this study we evaluated the linear isothermal Ribo-SPIA pre-amplification method for qPCR-based gene-expression analysis in cancer cell lines and commercially available reference samples, optimized the pre-amplification workflow, and used the method in a large clinical sample set.

First, we could clearly demonstrate that differential expression is preserved after pre-amplification and that no substantial bias is introduced. The fold-changes between pre-amplified samples were compared to those observed between non-amplified samples in the largest set to date (194 genes, 4 samples, 1164 data points), revealing an accurate preservation of relative transcriptome composition despite the pre-amplification process. This is in accordance with previously reported findings on smaller datasets using qPCR [10,26]. However, careful interpretation of the results is warranted in case of very small fold changes in gene-expression between samples. We further noticed that the observed bias (high ddCq) is mainly due to a lower pre-amplification efficiency for the region targeted with qPCR. Assays with a large difference in Cq-value before and after pre-amplification may thus need redesign. Further studies are required to investigate the potential relationship between various factors (including target localisation in the transcript) and the observed bias; if conclusive, guidelines might be developed for design of better qPCR assays to be used in pre-amplified products to further reduce the bias. Important to note is that the comparison of gene-expression of non-amplified samples with pre-amplified samples is not possible, which means that all samples analysed in the same expression study need pre-amplification. Moreover, since a sequence-specific pre-amplification bias has been recognized this technique is not suitable for splice-variant quantification or any other study that aims at the comparison of expression levels of two genes.

We also assessed the need of DNase treatment before and of purification after pre-amplification. The results obtained show that neither of these procedures is required. This is an important finding, especially in large-scale gene-expression studies, as both techniques are time-consuming and add a substantial cost to the experiments. Furthermore, DNase treatment may lead to a loss of material and of mRNA integrity due to the exposure of the RNA samples to a high temperature during heat inactivation required for many commercial DNases.

Monitoring RNA quality and using intact RNA is of critical importance to obtain reliable gene-expression data and to ensure reproducibility of the results [27,28]. In this study we assessed the RNA quality of 738 tumour samples before pre-amplification and evaluated the pre-amplification success by measuring the expression of two low abundant reference genes (*SDHA *and *HPRT1*). As expected, pre-amplification of highly degraded samples turned out to be unsuccessful. In addition, there was a negative correlation between the Cq-values of the reference genes and the RQI. A possible explanation for the imperfect negative correlation is the use of random primers in the RNA pre-amplification process, resulting in successful pre-amplification of partially compromised RNA samples.

As the tumour sample size is often very limited, the applied RNA pre-amplification procedure offers the possibility to perform large multicenter studies. This enabled us to establish and validate a robust prognostic multigene-expression signature in the largest neuroblastoma study cohort till now [19]. Moreover, the generated cDNA library is available for future qPCR-based gene-expression studies.

An additional advantage of the evaluated pre-amplification method is its potential usefulness to generate a sufficient nucleic acids concentration for use in ultra high-throughput qPCR systems. These systems operate with very low volumes and have the potential disadvantage of compromised detection sensitivity as only limited volumes of nucleic acids can be added. As the concentration of the pre-amplified material is very high, this technique may offer a solution and should be evaluated in future studies.

In conclusion, the results obtained from this study indicate that differential gene-expression is preserved after sample pre-amplification using the linear isothermal Ribo-SPIA pre-amplification method, that DNA is not co-amplified, that a pre-amplification clean-up step is not required, and that the pre-amplification product is free of enzymatic inhibitors. Application of this unbiased and straightforward pre-amplification technology offers a great advantage in terms of accessibility of material for diagnostic and prognostic work-up and enables large-scale qPCR gene-expression studies.

## List of abbreviations

Cq: quantification cycle; dCq: difference in quantification cycle or delta-Cq (measure for differential gene-expression); ddCq: difference in dCq or delta-delta-Cq (see additional file 3 for an example); HBRR: Human Brain Reference RNA; HGDNA: human genomic deoxyribonucleic acid; MAQC: MicroArray Quality Control; MNA: *MYCN *amplified; MNS: *MYCN *single copy; RNA: Ribonucleic acid; RQI: RNA quality index (determined by microfluidic capillary electrophoresis as a measure for RNA integrity); RT-qPCR: reverse transcription quantitative polymerase chain reaction; UHRR: Universal Human Reference RNA.

## Competing interests

The authors declare that they have no competing interests.

## Authors' contributions

JVM, SDR and JVS had substantial contributions to the conception, design, analysis, and interpretation of the data and the drafting of the manuscript for important intellectual content. JVM, SDR, EDS and NYG contributed to the wet lab work (RNA extraction, RNA quality control, RNA pre-amplification and qPCR) and interpretation of the data. KDP participated in the data analysis. FPT and SLF participated in the design and validation of the primers. ADP, FSP and JVS coordinated the study. All authors contributed to the revision of the manuscript for important intellectual content. All authors read and approved the final version of the manuscript.

## Supplementary Material

Additional file 1 **RDML file 1**. Primer sequences of the *MYCN *and *MYCN *regulated genes and raw data from the expression analyses on the 6 neuroblastoma cell lines. Click here for file

Additional file 2 **RDML file 2**. Primer sequences of the MAQC target genes and raw data from the expression analyses on the 4 MAQC reference samples. Click here for file

Additional file 3 **Supplemental Material and Methods**. Details on sample preparation, gene-expression analysis, formulas and raw data availability. Click here for file

Additional file 4 **Supplemental Figure S1**. Frequency distribution (left-axis) and cumulative frequency (right-axis) of the difference in quantification cycle value (dCq) induced by sample pre-amplification (x-axis) for 194 genes measured in the 4 MAQC reference samples. There is a clear sequence-specific pre-amplification bias, meaning that some sequences or parts of transcripts pre-amplify better than others. Click here for file

Additional file 5 **Supplemental Figure S2**. SPUD assay for the detection of enzymatic inhibitors in purified pre-amplified samples (P) and in non-purified pre-amplified samples (NP) with negative control (NC) and positives controls with known inhibitor (PC). Difference in Cq or delta-Cq (dCq) (NP or P vs. NC) < 1 indicates absence of enzymatic inhibitors. Click here for file

Additional file 6 **Supplemental Figure S3**. PCR efficiencies estimated with two different single curve efficiency algorithms, PCR Miner (red) and LinReg (blue). Efficiencies of purified (squares; 2 replicates) and non-purified (triangles; 2 replicates) pre-amplified samples for each gene are comparable indicating that non-purified pre-amplified samples do not contain inhibitors and amplify with the same PCR efficiency. Click here for file

Additional file 7 **Supplemental Figure S4**. Cumulative distribution plot of the delta-delta Cq (ddCq) before and after pre-amplification for 10 reference genes and 10 samples without purification of the pre-amplified product (black) and with purification of the pre-amplified product (grey). Each dot represents a ddCq-value between 2 samples before and after pre-amplification. Purification is not required for the preservation of differential expression. Click here for file

Additional file 8 **RDML file 3**. Primer sequences of *HPRT1 *and *SDHA *and raw data from the expression analyses on 738 neuroblastoma tumour samples. Click here for file
