# Supplementary material for: RNA pre-amplification enables large-scale RT-qPCR gene-expression studies on limiting sample amounts
Source: BMC Res Notes. 2009 Nov 25;2:235. doi: 10.1186/1756-0500-2-235 (PMC2789097; doi:10.1186/1756-0500-2-235)
Supplement: Additional file 3 — Supplemental Material and Methods. Details on sample preparation, gene-expression analysis, formulas and raw data availability. [file 1756-0500-2-235-S3.DOC]

**Supplemental Material and Methods**

*Sample preparation*

Total RNA extraction from 6 neuroblastoma cell lines (three *MYCN* single copy (MNS) neuroblastoma cell lines (1: GI-ME-N; 2: SK-N-AS; 3: SK-N-SH) and three *MYCN* amplified (MNA) neuroblastoma cell lines (1: IMR-32; 2: N206; 3: NGP)) and 738 fresh frozen neuroblastoma tumour samples was done in different laboratories by silica gel-based membrane purification methods (RNeasy Mini kit or MicroRNeasy kit, Qiagen), by phenol-based (TRIzol reagent, Invitrogen or Tri Reagent product, Sigma) or by chaotropic solution-based isolation methods (Perfect Eukaryotic RNA kit, Eppendorf) according to the manufacturer’s instructions and stored at - 80°C. All tumour samples were frozen immediately after removal from the patient and stored at - 80°C. Nucleic acid concentration was measured using the Nanodrop 1000 Spectrophotometer (Thermo Scientific).

Two commercial RNA samples were mixed (Universal Human Reference RNA (UHRR) from Stratagene and Human Brain Reference RNA (HBRR) from Ambion) to generate the four MAQC (MicroArray Quality Control) reference samples [1]: Reference 1/ 100% UHRR; Reference 2/ 100% HBRR; Reference 3/ 25% UHRR + 75% HBRR; Reference 4/ 75% UHRR + 25% HBRR.

This study was approved by the Ghent University Hospital Ethical Committee (EC2008/159).

*Assessment of sample purity and integrity*

In order to assess the quality of the RNA samples, we used 1 ng of each RNA isolate to perform capillary gel electrophoresis analysis (high sensitivity chips, Experion, software version 3.0, Bio-Rad) and establish an RNA quality index (RQI) ranging from 1 (poor RNA integrity) up to ten (high RNA integrity).

In order to assess the purity of the pre-amplified samples, we performed a PCR-based SPUD assay for the detection of enzymatic inhibitors in nucleic acid preparations [2]. In brief, a potato nucleic acid sequence of 101 bp (SPUD) at the concentration of 5000 molecules per µl, lacking homology with any other known human sequence, is amplified with specific primers. A mastermix is prepared including the SPUD sequence and primers and distributed over the wells. Subsequently, individual wells are supplemented with 10 ng RNA sample to be tested, or water (negative control) to determine the reference quantification cycle (Cq) value, or with a known inhibitor, such as heparin at a concentration of 0.3 to 0.4 U/ml (positive control). Consequently, Cq-values of the test samples are compared to the Cq-value of the negative control assay and a difference in Cq or delta-Cq (dCq) > 1 is used as a cut-off designating the presence of inhibitors.

*RNA pre-amplification and cDNA synthesis*

Starting from 5, 15, or 50 ng of total RNA, the WT-Ovation RNA Pre-amplification System (NuGEN) was used according to the manufacturer’s instructions, generating approximately 5 µg of cDNA (stored at – 20 °C), sufficient to measure more than 1000 target genes. Total non-purified pre-amplification product consisted of 42 µl at a concentration of approximately 800 ng/µl and was diluted to 6.5 ng/µl (corresponding to 1 ng/µl non-amplified cDNA). Briefly, this linear and isothermal pre-amplification method, randomly performed across the whole transcriptome, starts with ds cDNA synthesis followed by SPIA-based DNA pre-amplification to generate single-stranded cDNA pre-amplification product [3, 4].

In parallel the same RNA extracted from the neuroblastoma cell lines and the MAQC reference RNA samples were used for conventional cDNA synthesis. Briefly, 2 µg of total RNA was reverse transcribed using a mix of random and oligodT primers and iScript reverse transcriptase according to the manufacturer's instructions (Bio-Rad), and subsequently diluted with nuclease-free water (Sigma) to 5 ng/μl cDNA (total RNA equivalents) and stored at – 20 °C. Prior to the cDNA synthesis, a DNase treatment was performed using the RQ1 RNase-free DNase according to the manufacturer’s instructions (Promega).

*High-throughput real-time quantitative PCR-based gene-expression*

A quantitative polymerase chain reaction (qPCR) assay was designed for each gene and went through an extensive *in silico* validation process using BLAST and BiSearch specificity, amplicon secondary structure, SNP presence and splice variant analysis [5]. All amplicon lengths are comprised between 64 and 175 base pairs (mean: 98 bp). For all but eight primer pairs, the PCR amplification efficiency calculated using the LinReg single curve efficiency algorithm [6] was between 80% and 115% (mean efficiency: 89.9% (± 5.4 stdev)).

qPCR was done on a high-throughput 384-well-plate instrument (LC480, Roche). qPCR amplifications were performed in 7.5 µl containing 3.75 µl 2X SYBR Green I master mix (Roche), 0.375 µl forward and reverse primer (5 µM each), 1 µl nuclease-free water and 2 µl cDNA (10 ng). The cycling conditions consist of an initial step of 3 min polymerase activation at 95 °C and 45 cycles of 15 s at 95 °C and 30 s at 60°C, followed by a dissociation curve analysis from 60 °C to 95 °C. Duplicates were performed for all samples and the average of both Cq-values was used for calculations. No amplification curve was observed for NTCs (in duplicate) or the Cq-value was at least 10 cycles higher than the Cq-value of the samples.

*Calculation of the difference in delta-Cq-value (delta-delta-Cq or ddCq)*

The ddCq before and after pre-amplification should be zero and is calculated as follows:


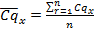
 (1)


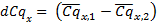
 (2)


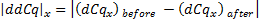
 (3)

where ‘n’ is the number of replicates (r), ‘x’ is ‘gene x’, ‘1’ and ‘2’ are ‘sample 1’ and ‘sample 2’, respectively and ‘before’ and ‘after’ are ‘before pre-amplification’ and ‘after pre-amplification’, respectively.

Example:

x = *TGFBI*

1 = SK-N-SH

2= NGP

|  | **sample** | 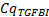 | 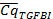 | 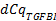 | 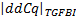 |
| --- | --- | --- | --- | --- | --- |
|  |  |  | (formula 1) | (formula 2) | (formula 3) |
| **before**  **pre-amplification** | SK-N-SH | 20.07 | 20.07 | -14.94 | 0.06 |
|  |  | 20.07 |  |  |  |
|  | NGP | 34.66 | 35.01 |  |  |
|  |  | 35.36 |  |  |  |
| **after**  **pre-amplification** | SK-N-SH | 23.02 | 22.96 | -14.88 |  |
|  |  | 22.90 |  |  |  |
|  | NGP | 37.61 | 37.84 |  |  |
|  |  | 38.07 |  |  |  |

*Data availability*

The experimental data is available in [additional files 1-2-8] in RDML (Real-time PCR Data Markup Language) format [7] according to the MIQE guidelines [8]. The primer sequences and target genes of each assay are available in [additional files 1-2-8] together with their corresponding RTPrimerDB ID. Detailed information about each assay record in the RTPrimerDB database is available in RDML format.

**References**

1. Shi L, Reid LH, Jones WD, Shippy R, Warrington JA, Baker SC, Collins PJ, de Longueville F, Kawasaki ES, Lee KY *et al*: **The MicroArray Quality Control (MAQC) project shows inter- and intraplatform reproducibility of gene expression measurements**. *Nat Biotechnol* 2006, **24**(9):1151-1161.

2. Nolan T, Hands RE, Ogunkolade W, Bustin SA: **SPUD: a quantitative PCR assay for the detection of inhibitors in nucleic acid preparations**. *Anal Biochem* 2006, **351**(2):308-310.

3. Dafforn A, Chen P, Deng G, Herrler M, Iglehart D, Koritala S, Lato S, Pillarisetty S, Purohit R, Wang M *et al*: **Linear mRNA amplification from as little as 5 ng total RNA for global gene expression analysis**. *Biotechniques* 2004, **37**(5):854-857.

4. Kurn N, Chen P, Heath JD, Kopf-Sill A, Stephens KM, Wang S: **Novel isothermal, linear nucleic acid amplification systems for highly multiplexed applications**. *Clin Chem* 2005, **51**(10):1973-1981.

5. Lefever S, Vandesompele J, Speleman F, Pattyn F: **RTPrimerDB: the portal for real-time PCR primers and probes**. *Nucleic Acids Res* 2009, **37**(Database issue):D942-945.

6. Ruijter JM, Ramakers C, Hoogaars WM, Karlen Y, Bakker O, van den Hoff MJ, Moorman AF: **Amplification efficiency: linking baseline and bias in the analysis of quantitative PCR data**. *Nucleic Acids Res* 2009, **37**(6):e45.

7. Lefever S, Hellemans J, Pattyn F, Przybylski DR, Taylor C, Geurts R, Untergasser A, Vandesompele J: **RDML: structured language and reporting guidelines for real-time quantitative PCR data**. *Nucleic Acids Res* 2009, **37**(7):2065-2069.

8. Bustin SA, Benes V, Garson JA, Hellemans J, Huggett J, Kubista M, Mueller R, Nolan T, Pfaffl MW, Shipley GL *et al*: **The MIQE guidelines: minimum information for publication of quantitative real-time PCR experiments**. *Clin Chem* 2009, **55**(4):611-622.
